# Licochalcone A Suppresses Renal Cancer Cell Proliferation and Metastasis by Engagement of Sp1-Mediated LC3 Expression

**DOI:** 10.3390/pharmaceutics15020684

**Published:** 2023-02-17

**Authors:** Tsai-Yi Tseng, Chien-Hsing Lee, Hsiang-Lin Lee, Chien-Yu Su, Cheng-Yen Kao, Jen-Pi Tsai, Yi-Hsien Hsieh

**Affiliations:** 1 Institute of Medicine, Chung Shan Medical University, Taichung 40201, Taiwan; 2 Division of Pediatric Surgery, Department of Surgery, Children’s Hospital of China Medical University, Taichung 404333, Taiwan; 3 School of Chinese Medicine, College of Chinese Medicine, China Medical University, Taichung 404333, Taiwan; 4 Department of Surgery, Chung Shan Medical University Hospital, Taichung 40201, Taiwan; 5 School of Medicine, Chung Shan Medical University, Taichung 40201, Taiwan; 6 Institute of Microbiology and Immunology, College of Life Sciences, National Yang Ming Chiao Tung University, Taipei 11221, Taiwan; 7 School of Medicine, Tzu Chi University, Hualien 970374, Taiwan; 8 Division of Nephrology, Department of Internal Medicine, Dalin Tzu Chi Hospital, Buddhist Tzu Chi Medical Foundation, Chiayi 62247, Taiwan; 9 Department of Medical Research, Chung Shan Medical University Hospital, Taichung 40201, Taiwan

**Keywords:** Licochalcone A, renal cell carcinoma, proliferation, migration, invasion, autophagy, Sp1, LC3B

## Abstract

Licochalcone A (LicA) is a strong anti-inflammatory, antioxidant, and anticarcinogenic substance that is useful against a variety of human malignancies. However, its precise mechanism in mediating the development of renal cell carcinoma (RCC) is not entirely understood. In this work, LicA was discovered to limit cell growth and survival, induce cell cycle arrest, promote autophagy and LC3B expression, and inhibit the migration and invasion of RCC cells. In addition, the proliferation, migration, and invasion inhibited by LicA were restored by the transfection of siRNA-LC3. The effects of LC3B on the metastatic phenotype of ACHN cells was enhanced with the overexpression of Sp1 or suppressed by inhibiting the phosphorylation of FAK and Src. Finally, LicA showed antitumor properties against RCC in an in vivo xenograft model. In conclusion, our study demonstrated the chemotherapeutic potential of LicA on proliferation, migration, invasion, and autophagy through the activation of LC3B expression, ultimately modulating FAK/Src signaling pathway-mediated Sp1 expression. These findings illustrate the novel role and molecular mechanisms of LicA against RCC cells.

## 1. Introduction

Renal cell carcinoma (RCC) is the most fatal genitourinary cancer and accounts for more than 90% of newly diagnosed kidney cancer cases. When RCC is detected at an advanced stage or with distant metastases, its median survival time and overall survival are constrained [1]. Although there have been developments in hormonal therapy and chemotherapy with interferon alfa and high-dose interleukin 2 (IL-2), limited improvements have been made because of low efficacy and high rates of toxicity [2,3]. Recently, however, therapies that target signaling pathways, such as immune checkpoint, tyrosine kinase (TKI), and mammalian target of rapamycin (mTOR) inhibitors, have steadily improved clinical results for advanced RCC [4,5]. Despite these encouraging advances, more work is needed to improve the limited median overall survival and objective response rates of RCC treatment using current therapies.

An intracellular degradative process called autophagy, which is characterized by an accumulation of autophagic vacuoles, is crucial in controlling the natural cell death that takes place during development. It is a crucial system for cellular survival when metabolic stress is present [6]. Autophagy has been linked to several cancers, including osteosarcoma, cervical cancer, breast cancer, and prostate cancer [6,7]. Furthermore, recent studies have demonstrated an intimate relationship between RCC and autophagy-related proteins, which could be a promising target for RCC treatment or for use as a marker indicative of tumor progression [8]. According to a recent study, the ability of RCC cell lines to proliferate could be inhibited by upregulating the expression of LC3, which is thought to be a signal for the beginning of autophagy because of its aggregation and localization on autophagosomes [7]. Another study showed that a phytochemical substance could induce autophagy to retard the migration and invasion of RCC cells [9].

The Src family tyrosine kinase (SFK, including Src) and focal adhesion kinase (FAK), known as the cytosolic nonreceptor tyrosine kinases, were the first proto-oncogene tyrosine kinases to be discovered [10]. Both have been reported to modulate critical signaling pathways for cell proliferation, survival, and metastatic phenotypes in multiple cancers, including breast cancer [11], colorectal cancer [12], non-small-cell lung cancer cells [13], and cervical cancer [14]. The way in which SFK and FAK regulate the malignant process is through interaction with tyrosine kinase receptors and steroid receptors as well as through transcription activation, leading to diverse biological functions such as cell survival, cell motility, and distant metastasis [10,15]. There is evidence that intervening in the function of FAK and Src kinase could inhibit invasion and migration in RCC [16].

Licochalcone A (LicA; C21H22O4) is a natural chalcone and phenolic which was isolated from the roots of the *Glycyrrhiza* species *G. glabra* and *G. inflata*, and widely used clinically in traditional Chinese medicine (TCM) [17]. LicA could also have potential as an antibacterial agent for the preservation of food [18]. Clinical practice has demonstrated that LicA has unique medicinal properties for reducing heat and toxins [19]. There is also evidence that LicA inhibits tumorigenesis and distant metastasis of cancer cells by inducing apoptosis [20] and autophagy [21], and arresting cell cycle progression [22], migration, and invasion [23]. Recently, LicA has been found to inhibit the invasion and migration of human hepatocellular carcinoma cell lines via suppression of the urokinase plasminogen activator [24]. Additionally, LicA could suppress the PI3K/Akt/mTOR signaling pathway, which would induce autophagy and apoptosis [25]. Although there have been rapid advances in treatment, the prognosis of advanced RCC, particularly with distant metastases, remains poor. Recent reports suggest that natural compounds, such as kaempferol [26] and thymoquinone [27] might inhibit the potential of human RCC cells to become malignant. This is promising in the development of new therapeutic approaches for RCC treatment. However, there is currently limited evidence for the antitumor activity of LicA in RCC. The purpose of this study was to ascertain the mechanism of action of LicA in relation to its antitumor effectiveness against RCC. 

## 2. Materials and Methods

### 2.1. Chemical Reagent and Culture Medium

Licochalcone A (LicA; BP0855) was purchased from Chengdu Biopurify Phytochemicals Ltd. (Chengdu, China). LicA was dissolved the DMSO solution and stock concentration was 100 mM. MTT, dimethyl sulfoxide (DMSO), 4,6-diamidino-2-phenylindole (DAPI) and Triton X-100 were purchased from Sigma-Aldrich (St. Louis, MO, USA). PF-753228 (FAK inhibitor) and PP2 (Src inhibitor) were purchased from BioVision Inc. (Milpitas, CA, USA). Penicillin/Streptomycin (P/S) reagent, fetal bovine serum (FBS), Dulbecco’s Modified Eagle Medium (DMEM)/F12 (DMEM/F12) Minimum Essential Media (MEM), RPMI1640 and 0.25% Trypsin-EDTA were purchased from Cytiva Life Sciences (Marlborough, MA, USA). Immobilon-E polyvinylidene difluoride (PVDF) membrane was obtained from Merck (Darmstadt, Germany). Lipofectamine 3000 Transfection Reagent, RNAiMAX Transfection Reagent and Acridine Orange (AO) dye were purchased from Invitrogen (Thermo Fisher Scientific, Waltham, MA, USA). Polymerase chain reaction (PCR) primers were obtained from Mission Biotech (Taipei, Taiwan). Anti-LC3 antibody was purchased from Novus Biologicals, Inc. (Littleton, CO, USA). Anti-Sp1 and anti-β-actin antibodies were purchased from Santa Cruz Biotechnology (Dallas, TX, USA). Anti-phosphoryl-FAK (p-FAK), total-FAK (t-FAK), phosphoryl-Src (p-Src) and total-Src (t-Src) antibodies were purchased from Cell Signaling (Danvers, MA, USA).

### 2.2. RCC Cell Culture

Human renal cancer 786-O and ACHN cell lines were purchased from Bioresource Collection and Research Center (Hsinchu, Taiwan) and cultured in RPMI-1640 medium (Cytiva, SH30011.02) and MEM medium (Cytiva, SH30193.04). The human proximal tubule epithelial HK2 cell line was incubated with DMEM/F12 medium (Cytiva SH30004.03). These cell culture media contained 10% FBS (Cytiva SH30071.02), NEAA solution (100X; Cytiva SH30238.01), P/S reagent (100X, Cytiva SV30010) and cells incubated at 37 °C (involved in 5% CO_2_) in a humidified atmosphere. RCC and HK-2 cells were subcultured when cells reached 70% confluence.

### 2.3. Cell Viability Assay 

Cell viability method by MTT assay previously report [28]. Briefly, 786-O (1.5 × 10^4^) and ACHN (4 × 10^4^) cells were cultured in a 24-well plate and treated with different concentrations of LicA for 24 and 48 h. Then, the 0.5 mg/mL MTT solution was added for 3 h at 37 °C. At the end of the reaction time, added the isopropanol to product the blue-violet crystals for 15 min. Cell growth rate was detected using the absorption wavelength at optical density (OD) 570 nm by spectrophotometer.

### 2.4. Cell Cytotoxicity Assay

Detection of cell cytotoxicity level was performed using the CytoscanTM-LDH Cytotoxicity assay kit (G Bioscience, St. Louis, MO, USA) according to the manufacturer’s manual. Briefly, RCC cells (8 × 10^3^/well) were seeded into a 96-well dish with triplicate sets of wells. For the LDH-positive control assay, the same assay was used as in the wells containing cells in triplicate. After incubation with LicA for 24 h, to the positive control wells was added 10 μL lysis buffer and these were then incubated for 45 min. Then, added the 50 μL supernatant combined with 50 μL reconstituted Substrate Mix to incubated at 37 °C for 20 min. At the end of the incubation period, 50 μL of Stop solution was added and OD absorbance was recorded at 490 nm using a microplate reader (BioTek, Winooski, VT, USA). The positive control value was calculated as 100% LDH content. The relative LDH expression was presented as the ratio of the value of the experimental group and the value of the positive control.

### 2.5. Colony Formation Assay

786-O and ACHN cells (2 × 10^3^/well) treated with LicA were seeded into a 6-well culture dish for 7 days, these colonies became visible; then, they were fixed with 4% paraformaldehyde for 10 min and stained with crystal violet reagent (1:20) for 20 min. Counting of the number of crystal violet colonies by using an inverted microscope (Nikon Instruments, Inc., Melville, NY, USA).

### 2.6. Cell Cycle Phase

After treatment with various concentration of LicA in a 6 cm culture dish for 24 h, cell pellets were collected and washed with PBS, then the supernatant was removed and 75% alcohol was slowly added to the pellets on ice at −20 °C for 72 h. Next, added propidium iodide (PI)/Triton-100 solution (0.02 mg/mL RNAase and 0.1% Triton-X100) was followed by staining in the dark for 20 min. The results were detected for Muse^®^ Cell Analyzer (Millipore, Hayward, CA, USA).

### 2.7. In Vitro Cell Migration and Invasion

Determination of cell migration and invasive abilities was carried out as in a previous report [29]. After transfection with 0.1 μM siRNA-LC3 [30] or 1 μg Sp1 plasmid [31] for 24 h, then treated with LicA for another 24 h. Then, RCC cells were added into the upper 48-well chemotaxis chambers with an 8 μm pore size membrane filter (SCWP04700; Merck KGaA, Darmstadt, German), which was pre-coated without (migration) or with (invasion) Matrigel (Corning Life Sciences, Durham, NC, USA) in an FBS-free medium. In the low chambers (including 10% FBS + 90% culture medium). After incubation for 16 h (migration) or 20 h (invasion), the migrating or invading cells were incubated with methanol for 5 min and then stained with Giemsa solution (1:20) for 30 min. The migration or invasion ability of cells was counted using a microscope (Nikon Instruments, Inc., Melville, NY, USA). ×100 magnification.

### 2.8. siRNA and Plasmid Transfection

786-O and ACNH cells were transfected with 100 nM si-LC3 by Lipofectamine™ RNAiMAX Transfection Reagent as following a previously report [30]. The construct overexpression plasmid containing the Sp1 sequence was kindly gift from the Dr. Shun-Fa Yang laboratory (Chung Shan University, Taichung, Taiwan). ACHN cells were transfected with Sp1 plasmid for 24 h using the Lipofectamine^®^ 3000 transfection reagent. After transfection with siRNA-LC3 or Sp1 plasmid, LicA (12.5 μM) was added for another 24 h. The total protein was extracted, and the expression detected using Western blotting.

### 2.9. Western Blotting

Extraction of total lysate from LicA-treated cells and quantified protein using a Bradford assay (Thermo Fisher Scientific Inc., Waltham, MA USA). Total protein (20 μg) was subjected to electrophoresis by using 10–12% SDS-PAGE for 1.5 h and transferred to PVDF membrane with transfer buffer for 1 h. The PVDF membranes incubated with blocking buffer (5% skim milk) for 1 h. Primary antibodies added and placed at 4 °C for overnight. Antibodies against the anti-LC3B (1:1000), anti-Sp1 (1:1000), anti-β-actin (1:10,000), phosphoryl-FAK (p-FAK, 1:1000), total-FAK (t-FAK, 1:1000), phosphoryl-Src (p-Src, 1:1000) and total-Src (t-Src, 1:1000). Next, secondary antibodies against horseradish peroxidase-conjugated antibodies (1:10,000) were added for 1 h, then washed with PBS twice per 5 min at room temperature. Finally, the result was treated with chemiluminescent reagents (Merck Millipore, Burlington, MA, USA) to detect the indicated protein expression by using an ImageQuant LAS 4000 Mini (Cytiva Life Sciences, Marlborough, MA, USA).

### 2.10. Real-Time Reverse Transcription Polymerase Chain Reaction (qRT-PCR)

The total RNA by using TRIzol reagent (Invitrogen, Carlsbad, CA, USA) and equal amounts of RNA (0.5 μg/μL), reversed to cDNA using a GoScript RT System kit with oligo dT (Promega, Madison, WI, USA). Then, the mRNA levels of Sp1 expression were quantified using GoTaq qPCR Master Mix (Promega, Madison, WI) in an Applied Biosystems 7700 Real-time (RT) PCR System (Applied Biosystems, Waltham, MA) as per the manufacturer’s protocol. GAPDH was used as a as an internal reference gene for mRNA expression. Gene expression levels were calculated using the 2^–ΔΔCt^ method. The PCR primer sequences were Sp1-Forward: 5′-TGGCAGCAGTACCAATGGC-3′; Sp1-Reverse: 5′-CCAGGTAGTCCTGTCAGAACTT-3′. GAPDH-Forward: 5′-CATCATCCCTGCCTCTACTG-3′; and GAPDH-Reverse: 5′-GCCTGCTTCACCACCTTC-3′.

### 2.11. Acidic Vesicular Organelle Staining

RCC cells (4 × 10^5^ cells/well) were treated with LicA. After 24 h, RCC cells were exposed to AO staining reagent (1 μM) and Hoechst 33342 (2 μM) for 30 min as followed previously report [30]. Subsequently, cells were washed with PBS three times, and fluorescence intensity used to analyze the AO-stained cells using ImageXpress Pico Automated Cell Imaging System (Molecular Devices, LLC, San Jose, CA, USA). Quantification of the fluorescence intensity were measuring the CellReporterXpress Software (Molecular Devices, LLC, CA, USA).

### 2.12. Observation of LC3 by Immunofluorescence Staining 

The immunofluorescence staining for LC3 expression was followed the previously reports [32]. RCC cells incubated with 8-well Lab-Tek chambered coverglass (#155411; Thermo Fisher Scientific Inc.) and added the LicA for 24 h. RCC cells were fixed in 4% formaldehyde for 30 min, added with 0.1% Triton X-100 for 10 min and incubated with 2% BSA (bovine serum albumin) for 1 h at 37 °C. Next, incubated with the anti-LC3 antibody (1:100) at 4 °C overnight, and mouse or rabbit IgG antibody (1:500) was added for 20 min at room temperature. DAPI reagent was added the LicA-treated RCC cells for 10 min and mounted using a mounting buffer on a chambered coverglass. The observed fluorescence images were acquired under a confocal microscope (Leica, Mannheim, Germany).

### 2.13. Chromatin Immunoprecipitation (ChIP)

ACHN cells (2 × 10^6^ cells) were treated with LicA 25 μM for 24 h. Then, cells were crosslinked with 4% paraformaldehyde for 10 min; the reaction was terminated by using glycine (125 mM) for 5 min. Cells were then put on ice and washed with PBS three times, the lysate extracted by using a NETN buffer and the lysed cells were sonicated (60 Hz) in ice for 10 min. The pellets were removed and the collected supernatant (0.5 mg) was pre-absorbed by protein A/G beads (60 uL). Subsequently, the mixture was immunoprecipitated with the primary antibody Sp1 (1 μg/mL) or with rabbit immunoglobulin G (1 μg/mL) overnight. On the next day, the mixture was centrifuged and the magnetic beads collected, then rinsed three times with lysis buffer, three times with LiCl buffer, and three times with Tris-EDTA buffer (pH 8.0). The sample was removed the RNAase and protein content by using RNase A (0.2 mg/mL). and proteinase K (10 mg/mL). A DNA purification kit was used and nuclease-free water added to collect the DNA. The chromatin immunoprecipitation (ChIP) primers used were as follows: LC3B-I forward: 5′-TCGGATTTGCCCCATGTCCC-3′; LC3B-I reverse: 5′-TGAGGTGACGGTTGTGGGC’; LC3B-II forward: 5′-TGCTGGGTTCCGCCAC-3′; and LC3B-I reverse: 5′-ATCTCCTCAGCCCGCAG′. 

### 2.14. In Vivo Tumorigenesis Assay

Female BALB/c nude mice (four weeks old; National Laboratory Animal Center (Taipei, Taiwan)) were used, following the in vivo xenograft mice model [33]. This study was approved by the Animal Institutional Animal Care and Use Committee of Chung Shan Medical University (IACUC number: 1910; 2017/06/08). Mice were divided into two nude mouse groups (n = 5/each group): the control group and the LicA group. Control group: Mice were injected with 786-O cells (100 µL; 1 × 10^7^ cells/mL) into the right flank of the nude mice. LicA group: Mice were injected with 786-O cells (100 µL; 1 × 10^7^ cells/mL) into the right flank of the nude mice. After tumor formation 7 days later, mice were orally administered LicA (20 mg/kg) twice a week for 18 days. The tumor growth rate of these mice was measured every 3 days, beginning after tumor injection. The tumor volume formula as follows: L1 × L^2^ × 0.523. The animal experiment was terminated at 18 days. All mice were sacrificed with CO_2_ and the tumor excised from the right flank of the nude mice. Tumor tissues were photographed, weighed and fixed with 10% formalin. 

### 2.15. Detection of Putative Transcription Factor Binding Sites of LC3 Promoter

The transcription factors that bind to LC3B promoter were predicted using PROMO version 8.3 (https://alggen.lsi.upc.es/cgi-bin/promo_v3/promo/promoinit.cgi?dirDB=TF_8.3, accessed on 15 January 2012) as previously described [34]. The human LC3B promoter was used for transcription factor search and found that transcription factor Sp1 with the highest target score was used for further study.

### 2.16. Statistical Analysis

Quantifications of Western blots were performed using ImageJ 3.1 software. The experimental data were analyzed using Graphpad Prism (6.0) and SPSS (12.0) statistical software. The results are shown as mean ± standard deviation (SD) and analyzed using one-way ANOVA (analysis of variance) was determined by Dunnett post hoc test. * and ** indicate *p* < 0.05 and *p* < 0.01 (statistically significant). # indicates *p* < 0.05 (statistically significant), compared with the LicA-treated group.

## 3. Results

### 3.1. LicA Inhibited the Growth and Induced Cell Cycle Arrest of 786-O and ACHN Cells

**Figure 1:**
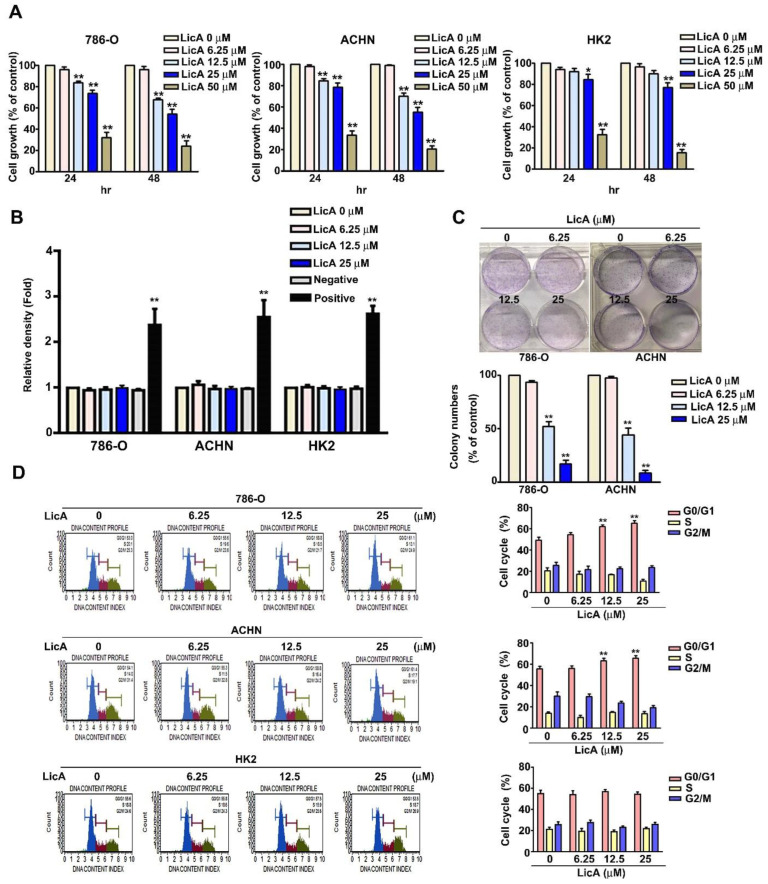
The effects of LicA on RCC cell proliferation. (**A**) For 24 and 48 h, different doses of LicA (0, 6.25, 12.5, 25, and 50 µM) were incubated with RCC (786-O, ACHN) and normal renal tubular (HK2) cells. Cell proliferation was evaluated using MTT assay. (**B**) LDH assay and spectrophotometry were used to test for cell toxicity in RCC cells (786-O and ACHN) and normal HK2 cells after exposure to different doses of LicA (0, 6.25, 12.5, and 25 µM) for 24 h (positive control with 1% Triton X100). (**C**) The proliferation of 786-O and ACHN cells was then determined by harvesting the cells with different LicA concentrations (0, 6.25, 12.5 and 25 µM) and counting the number of colonies. (**D**) PI staining and flow cytometry were used to investigate the cell cycle distribution of RCC cells (786-O, ACHN) and normal HK2 cells that had been exposed to different doses of LicA (0, 6.25, 12.5, and 25 µM). The results are shown as the mean ± SD of at least three different tests. * *p* < 0.05; ** *p* < 0.01, compared with that of the untreated control (0 µM).

First, we exposed 786-O and ACHN cells to LicA at a range of concentrations (0, 6.25, 12.5, 25, and 50 μM) for 24 and 48 h in order to study the cytotoxicity of LicA. We found that treatment with LicA resulted in a dose-dependent, significant reduction in cell growth, as measured by MTT assay (Figure 1A), and cell proliferation, as measured by colony formation assay, related to the 786-O and ACHN cells (Figure 1C). Interestingly, LicA was found to have few cytotoxicity effects on the cell growth of normal renal tubular cells (HK2) at concentrations of 25 µM for 24 h; however, LicA at 50 µM for 24 and 48 h had greater toxicity to the HK2 and RCC cells (Figure 1A). Therefore, the concentration of LicA used throughout this study was no more than 25 µM. Furthermore, LDH assay detected no LicA toxicity to RCC cells (786-O, ACHN) and normal HK2 cells (Figure 1B).

To explore the cell cycle distribution of RCC cells (786-O, ACHN) and normal HK2 cells in order to investigate the potential impacts of LicA on cell cycle arrest, RCC and HK2 cells were exposed to LicA at different concentrations (0, 6.25, 12.5, and 25 μM) for a period of 24 h. At concentrations of 12.5 and 25 µM, there was a signifciant increase in the percentage of LicA-treated 786-O and ACHN cells arrested at the G0/G1 phase (Figure 1D), but not affect the cell cycle distribution of normal HK2 cells (Figure 1D). Cells from the 786-O, ACHN and HK2 cell lines were also treated with LicA at different concentrations (0, 6.25, 12.5, and 25 μM) for 24 h to see if this might cause cell death in RCC cells and normal HK2 cells. Using the Annexin V/PI double stained test with flow cytometry, we discovered that LicA had no impact on the apoptosis of 786-O, ACHN and HK2 cells (Supplementary Figure S1). These results suggested that LicA could inhibit proliferation of 786-O and ACHN cells by inducing cell cycle arrest in LicA-treated RCC cells.

### 3.2. LicA Suppressed Invasion and Migration of 786-O and ACHN Cells

**Figure 2:**
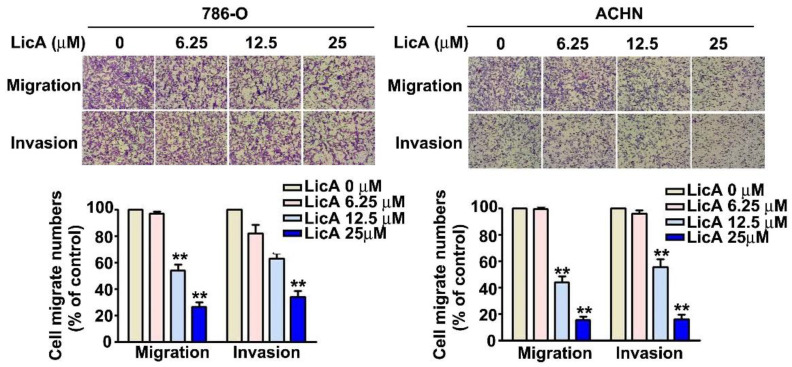
The effects of LicA on the invasion and migration of RCC cells. After being exposed to different concentrations of LicA (0, 6.25, 12.5, and 25 µM) for 24 h, 786-O and ACHN cells were tested for migration and invasion utilizing migration and invasion assays. Giemsa stain was used to stain cells on the lower surface of the Boyden chamber, and the cells were then photographed at 400× under a light microscope. The quantity of migrating or invaded cells is presented as a histogram chart in the lower panel. The results of at least three separate experiments are reported as means ± SD. ** *p* < 0.01, compared with that of the untreated control (0 µM).

Investigations were conducted to discover how LicA affected RCC cell invasion and migration. We investigated in vitro migration and invasion in 786-O and ACHN cells after 24 h of incubation with different doses of LicA (0, 6.25, 12.5, and 25 μM). There was a significant, dose-dependent reduction in the migration and invasion of both LicA-treated 786-O and ACHN cells at 12.5 and 25 µM (Figure 2). These results showed that LicA could inhibit migration and invasion of human RCC cells.

### 3.3. LicA Induced Autophagy of 786-O and ACHN Cells

**Figure 3:**
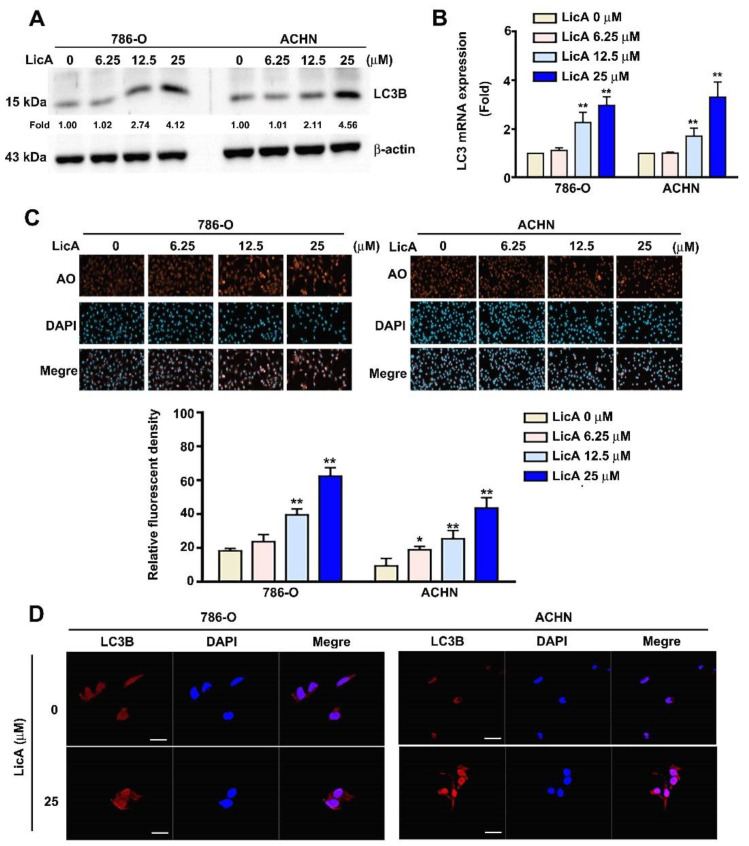
The effects of LicA on the expression of LC3B and autophagy in RCC cells. LicA was applied to 786-O and ACHN cells in a range of concentrations (0, 6.25, 12.5, and 25 µM). (**A**) Western blotting was utilized to detect LC3B in total protein lysates from treated cells, with β-actin serving as a loading control. (**B**) Total RNA was extracted from the cells treated with LicA and subjected to RT-PCR. (**C**) Using the relative fluorescence intensity of the AO (red) and DAPI reagents as counterstains, the effects of LicA on autophagy were measured. AO cell quantification was evaluated using flow cytometry. (**D**) The cell nuclei were counterstained with DAPI solution after the cells had been fixed and immunostained with antiLC3B antibody (red). The data represent the mean ± SD of at least three separate experiments. * *p* < 0.05, ** *p* < 0.01, compared to untreated control (0 µM). Scale bar = 50 μm.

Cells from the 786-O and ACHN lines were cultured with different doses of LicA (0, 6.25, 12.5, and 25 μM) for 24 h to observe whether LicA could trigger autophagy in RCC cells. Western blotting and RT-PCR assay (Figure 3A,B) revealed a dose-dependent increase in the expression of LC3B protein and mRNA, which was indicative of autophagy. The first stage of autophagy is the production of double-membrane vesicles known as autophagosomes. These vesicles then change into acidic, single-membrane autophagosomes and fuse with lysosomes to generate autolysosomes. Acidic vesicular organelles then form as a result of this process, which is accompanied by an increase in the acidity of the lumen. The relative fluorescence intensity of the 786-O and ACHN cells rose in a dose-dependent manner, as measured using a flow cytometry experiment following acidic organelle and immunofluorescence staining (Figure 3C). Additionally, immunostaining and confocal fluorescence microscopy indicated that LicA significantly increased the expression of LC3B of 786-O and ACHN cells at a concentration of 25 µM (Figure 3D). These results indicate that LicA induces autophagy through enhanced expression of LC3B in RCC cells.

### 3.4. Downregulated Expression of LC3B Enhanced the Malignant Potential of 786-O and ACHN Cells

**Figure 4:**
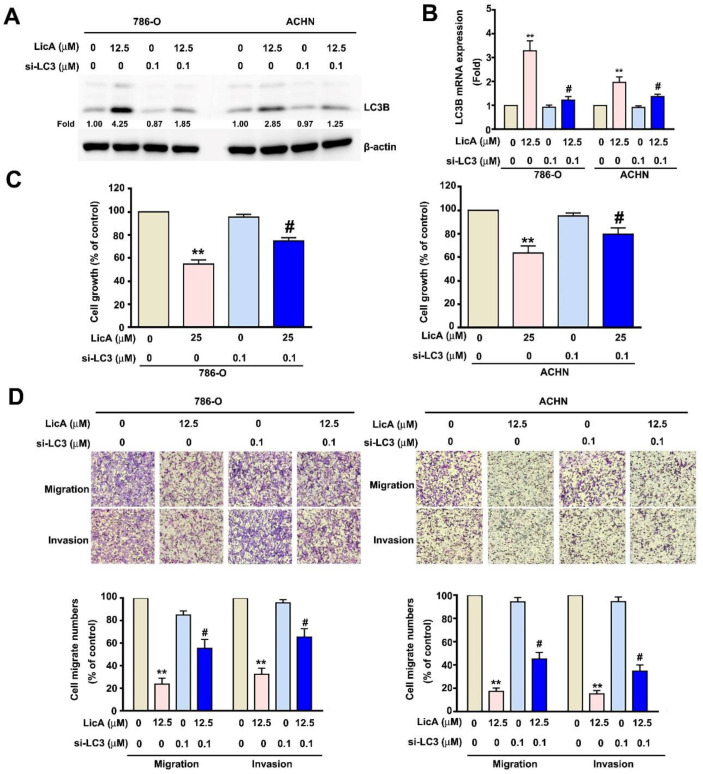
Effects of LC3B on RCC cell invasion, migration, and viability. After pretreatment with si-LC3 for 6 h, 786-O and ACHN cells were incubated with LicA (0 and 12.5 or 25 µM) for 24 h. (**A**,**B**) Western blotting and RT-qPCR were used to examine the expression of the protein of LC3B and total RNA. (**C**) The MTT assay was used to measure cell viability. (**D**) A migration assay and a Matrigel-invasion experiment were used to detect migration and invasion. In the lower panel, histograms representing the quantity of migrating or invaded cells are displayed. The data represent the mean ± SD of at least three separate experiments. # *p* < 0.05 or ** *p* < 0.01, compared to untreated control (0 µM).

There is some evidence that autophagy has a metastasis-inhibiting function in various tumor cells [35,36]. We set out to determine whether LC3B-induced autophagy was involved in the inhibition of RCC cell migration and invasion by LicA. To further understand the connection between LicA and autophagy, 786-O and ACHN cells were transfected with si-LC3B. Next, the si-LC3-transfected cells were treated to LicA (12.5 or 25 μM) for 24 h before being compared to unexposed cells. Western blotting and RT-PCR demonstrated that both the exposed and control si-LC3-transfected 786-O and ACHN cells expressed significantly less mRNA as well as LC3B in protein (Figure 4A,B). Additionally, MTT assay suggested that transient transfection with si-LC3 and treatment with 25 µM LicA resulted in reversed cell viability (Figure 4C). The migration and invasion assays found that 786-O and ACHN cells incubated with 12.5 µM LicA and transiently transfected with si-LC3 exhibited increased migration and invasion, compared to those without si-LC3 (Figure 4D). These results again demonstrated the antiproliferative and antimetastatic effects of LicA through the modulation of LC3B expression.

### 3.5. LicA Increased the Expression of LC3B by Inhibiting the Activation of Sp1 in ACHN Cells

**Figure 5:**
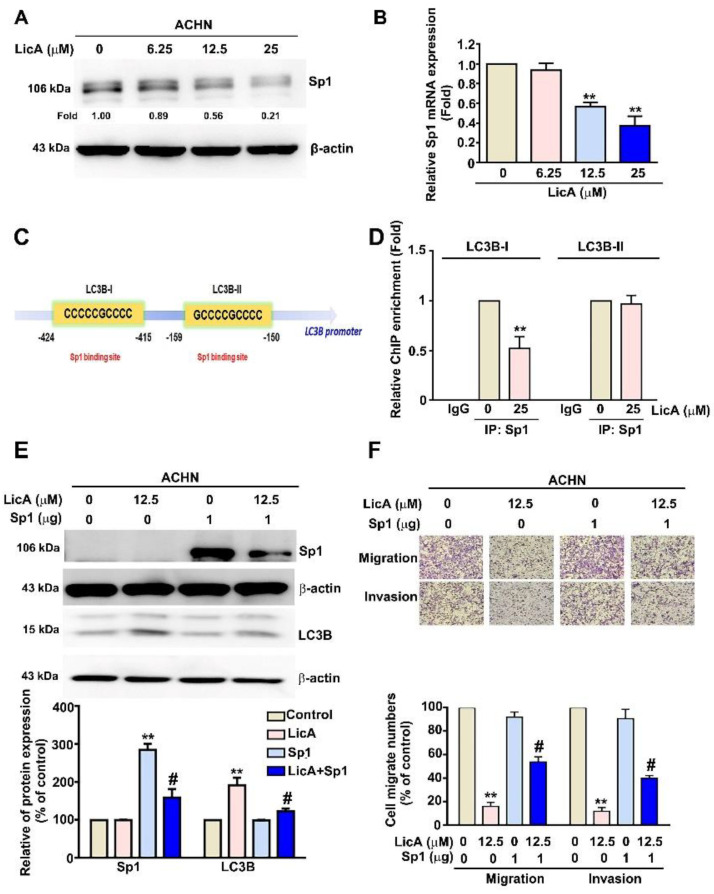
LicA inhibits cell migration and invasion by Sp1-targeted LC3B expression in RCC cells. ACHN cells were incubated with various concentrations of LicA (0, 6.25, 12.5, and 25 µM) for 24 h. Sp1 expression was verified using (**A**) Western blotting with β-actin used as a loading control and (**B**) RT-qPCR assay with GAPDH used as a loading control. (**C**) The transcription factor binding site of Sp1 for LC3B promoter. (**D**) Cells were treated with 25 µM and immunoprecipitated with antiSp1 or control IgG and then assessed using ChIP assay (shown as relative quantitative results). (**E**) The Sp1 plasmid was subsequently transfected into ACHN cells for 6 h, which were then exposed to 0 and 12.5 µM of LicA for 24 h. Western blotting was utilized to analyze the expression of the Sp1 and LC3B proteins, with β-actin serving as a loading control. (**F**) Using a migration assay and a Matrigel-invasion experiment, migration and invasion were assessed. The quantity of migrating or invaded cells is represented as a histogram chart in the lower panel. The data represent the mean ± SD of at least three separate experiments. ** *p* < 0.01, compared to untreated control (0 µM); # *p* < 0.05, compared to LicA-treated alone.

**Figure 6:**
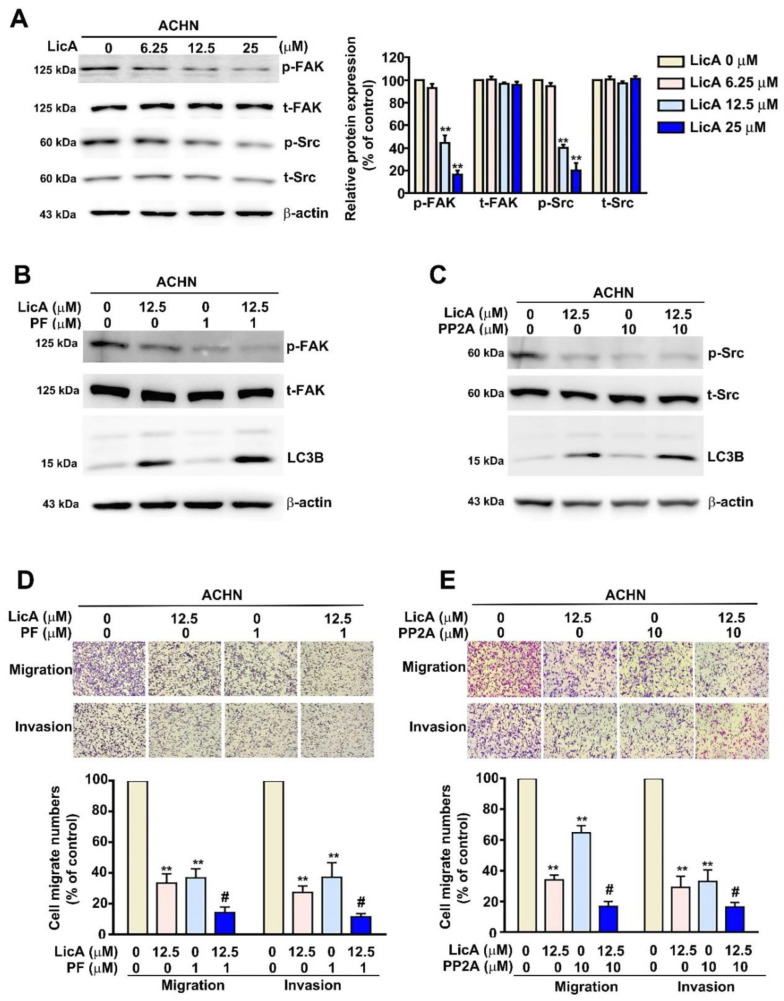
LicA inhibits metastasis via the FAK/Src signaling pathway of RCC cells. Different LicA concentrations (0, 6.25, 12.5, and 12.5 µM) were applied to ACHN cells, and the cells were then left to react for 24 h. (**A**) Western blotting was utilized to examine the expression of p-FAK, t-FAK, p-Src, and t-Src in cell lysates. β-actin served as a internal control. The ACHN cells were treated with 12.5 µM LicA in the presence or absence of (**B**) a FAK inhibitor (PF) or (**C**) an Src inhibitor (PP2) for 24 h. The cell lysates were examined using Western blotting to measure the protein levels of p-FAK, t-FAK, and LC3B. (**D**,**E**) Next, migration and Matrigel-invasion assays were used to assess migration and invasion. Data represent the mean ± SD of at least three separate experiments. ** *p* < 0.01, compared to un-treated control (0 µM); # *p* < 0.05, compared with LicA-treated alone.

Using the PROMO system (TRANSFAC, version 8.3), we found Sp1 to be the transcription factor binding site for the LC3B promoter region. Previous studies found evidence for Sp1 overexpression in a variety of malignancies [37]. Using Western blotting and RT-PCR assays, we found a significant, dose-dependent reduction in Sp1 protein and mRNA expression in ACHN cells treated with LicA at various concentrations (Figure 5A,B). ChIP assays were performed to clarify the involvement of LC3B-I and LC3B-II in LicA-induced inhibition of the binding of Sp1 to the LC3B transcription. These showed that when ACHN cells were treated with 25 µM LicA, the capacity of Sp1 to bind to the LC3B-I promoter region, but not to the LC3B-II promoter region, was significantly decreased (Figure 5D and Figure 6C). Using Western blotting, we found that cells transfected with Sp1 plasmid exhibited significantly decreased expression of LC3B protein, as well as increased migration and invasion, compared with ACHN cells treated with 12.5 µM LicA alone (Figure 5E,F). These results indicated that LicA increases LC3B expression by decreasing the binding of Sp1 to the LC3B-I binding site of the LC3B promoter region.

### 3.6. LicA Inhibited Tumorigenesis and Metastasis via the FAK/Src Signaling Pathway in ACHN Cells

Previous studies demonstrated that the FAK/Src signaling pathway is involved in the tumorigenic and metastatic processes [38]. We investigated whether LicA could affect RCC progression through this pathway. Western blotting and RT-PCR assays showed a significant, dose-dependent reduction in the protein and mRNA expression of phosphorylated FAK and Src in ACHN cells treated with various concentrations of LicA (6.25, 12.5, and 25 µM) (Figure 6A). To confirm that LicA-induced autophagy and antimetastasis occurred through the FAK/Src signaling pathway, we introduced 12.5 µM LicA-treated ACHN cells to PF-562271 (a FAK inhibitor; 1 μM) and PP2 (an Src inhibitor; 10 μM). We found that LC3B protein expression increased significantly, together with markedly decreased migration and invasion, compared with ACHN cells treated with LicA, PF, or PP2 alone (Figure 6B,C). These results indicated that LicA induces autophagy and suppresses metastasis by suppressing the activation of the FAK/Src signaling pathway.

### 3.7. Antitumorigenesis of LicA in 786-O Xenografts: In Vivo Animal Assay

**Figure 7:**
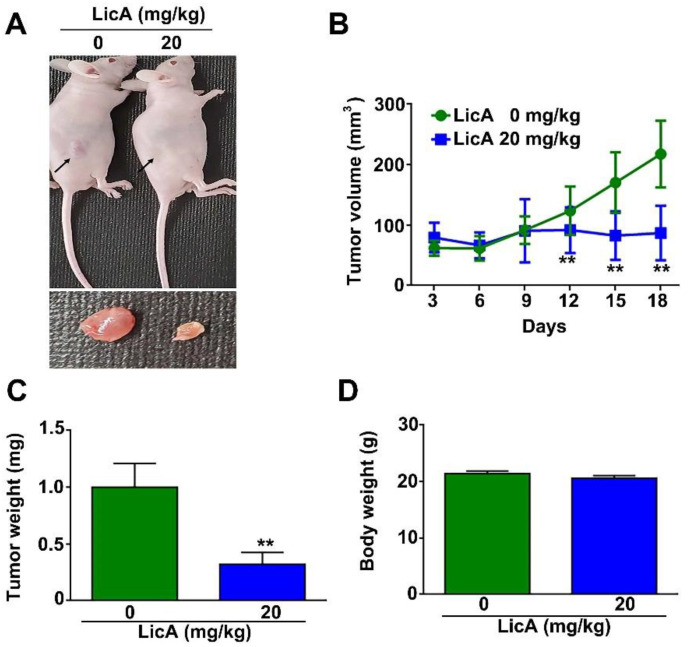
LicA suppresses tumor growth of RCC xenografts in vivo. (**A**) Representative images of the tumor tissues of the LicA-treated group and control group. (**B**) The tumor volume growth curve (mm^3^). (**C**) Tumor weight and (**D**) body weight changes in mice in each group during treatment. N = 5 in control and LicA-treated groups. ** *p* < 0.01, compared to control group.

As previously reported in relation to application in cervical cancer cells, we chose 20 mg/kg as the experimental dose in order to exert an effective antitumor effect and avoid toxicity to organs or blood biochemical analyses [25]. We subcutaneously injected 786-O xenografts in female nude mice. The image shows that LicA (20 mg/kg) treatment significantly suppressed tumor growth in the LicA-treated group (Figure 7A). LicA-treated mice exhibited an approximate 78% reduction in tumor growth on day 18 compared to the control (Figure 7B). There was a reduction in tumor weight (Figure 7C), but no significant differences in total body weight (Figure 7D). These results demonstrated that LicA can suppress the RCC tumorigenesis in vivo.

## 4. Discussion

Although RCC mortality has decreased as newly developed therapies have become available, survival is poor after progression with distant metastasis [39]. In recent decades, treatments against advanced and distant metastatic RCC have evolved from traditional surgery and chemotherapy to targeted therapies [5]. RCC has been treated with phytochemicals with pharmacological effects, including antioxidant, anti-inflammatory, and antineoplastic characteristics. [40,41]. Kaempferol, a polyphenol belonging to the flavonoid subgroup, exerted antiproliferative and antimetastasis effects in 786-O cells by attenuating metalloproteinase-2 (MMP-2) expression through the downregulation of the Akt and FAK signaling pathways [26]. Thymoquinone also inhibited migration and invasion in 786-O cells in a xenograft model by downregulating MMP-2 and the urokinase-type plasminogen activator (μPA). It also achieves this by reducing the adhesion of cells to type I and IV collagen via the suppression of the phosphorylation of PI3K, Akt, Src, and paxillin [27]. Through the Src/Ras/Raf/ERK signaling pathway, isothiocyanate, another natural chemical, significantly inhibited proliferation, induced apoptosis and cell cycle arrest, and prevented metastasis of 786-O cells [41]. It is well known that many phytochemicals exhibit pharmacological activities against RCC; for example, thimoquinone and kaempferol are natural compounds which have inhibitory effects on human RCC cells through different signaling pathways, by suppressing RCC cell proliferation and metastasis. Similarly, our results indicated that LicA has antitumor and antimetastasis effects on human RCC cells, but unlike other reports, we found that LicA induced LC3B expression through modulation of the FAK/Src/Sp1 signaling pathways. Therefore, LicA may highlight the possibility of another substance having novel therapeutic potential against human RCC through a unique pathway.

By inducing apoptosis through downregulation of Sp1 expression and downstream protein expression, LicA was shown to inhibit the growth of oral squamous cell carcinoma in a dose- and time-dependent manner [42]. By interfering with the mitogen-activated protein kinases (MAPK; ERK, JNK, and p38) and producing oxidative stress, LicA demonstrated significant effects on stopping cell cycle progression at the G2/M transition and triggering apoptosis in human gastric cancer [43,44]. By modulating the phosphorylation of p38 MAPK, LicA could induce the mitochondria-dependent intrinsic apoptotic signaling pathway in osteosarcoma cells [33]. In addition to causing apoptosis in cancer cells, LicA could cause cell cycle arrest during the G2/M phase in hepatoma HepG2 cells [20] or at the G0/G1 and G2/M phases in U87 glioma cells by modulating the expression of cyclins and cyclin-dependent kinases and inducing the endoplasmic reticulum stress-related proteins [22]. In non-small cell lung cancer H460 and A549 cells, LicA could suppress migration and invasion through a reduction in MMP-1 and MMP-3 expression. It does so by inhibiting the Akt pathway and its downstream transcription factor Sp1, in addition to inducing cell cycle arrest at the G2/M phase [23,45]. Recently, we discovered that LicA might prevent human hepatocellular carcinoma cells from migrating and invading through decreased production of μ-PA and inhibition of the JNK/MMK signaling pathway [24]. Taken together, although LicA did not affect apoptosis of 786-O and ACHN cells, this study provides evidence that it could suppress cell growth, proliferation, and arrest the cell cycle at the G0/G1 phase.

Along with Src family kinases, the active FAK forms a close-knit binary complex that can phosphorylate other substrates and activate a variety of intracellular signaling pathways to control a range of cellular processes, from cell survival and motility to distant metastasis. [10,15]. Src expression was substantially linked to tumor recurrence in samples of human colorectal malignancies and during clinical follow-up, and tumors that expressed both FAK and Src had a noticeably shorter time to recurrence. [46]. In tamoxifen-resistance breast cancer cells, activating Src expression could promote increased FAK phosphorylation and result in increased cell migration [47]. Another study of triple-negative breast cancer showed a more aggressive phenotypic transformation with enhanced FAK/Src activation through COL8A1 overexpression [11]. Arecoline, a potential carcinogen, and Timosaponin AIII, a type of steroid saponin, were found to exert either pro-carcinogenic or anti-metastatic effects on lung cancer cells by regulating the expression of MMPs through the EGFR/Src/FAK and ERK/Src/FAK signaling pathways [13,48]. In RCC, one study showed that when the FAK function was reduced, RCC cells would exhibit reduced migration and invasion and slower tumor growth [16]. Natural triterpene, a substance purified from medicinal mushrooms, demonstrates anti-cancerous effects by suppressing the promoter activities and downregulation of MMP-7 expression along as well as inhibiting the Src/FAK-paxillin signaling pathway [49]. Recently, the mTOR inhibitor everolimus was shown to suppress lung metastasis in 786-O RCC cells by reducing FAK/Src phosphorylation [4]. Taken together, activation of Src/FAK plays an important role in regulating the malignant phenotype of multiple cancers, including RCC. However, there was limited evidence demonstrating LicA’s effects on RCC by modulating autophagy. This is the first study to confirm that LicA could downregulate the phosphorylation of FAK/Src to induce autophagy and inhibit 786-O and ACHN RCC cell line invasion.

Specificity protein 1 (Sp1) is a ubiquitous factor belonging to the family of the transcription factors that could interact with promoter elements [50]. There is evidence that Sp1 controls genes involved in cell differentiation, the cell cycle, and apoptosis, which affect the development of numerous cancer cells. [51]. One study has shown that vascular endothelium growth factor could be suppressed by inhibiting the binding of Sp1 to the factor’s promoter and then blocking the activation of the c-Src/FAK signaling pathway [52]. By reducing Sp1 expression, another study showed reduced cell proliferation and increased apoptosis in multiple myeloma cells [53]. Recently, LicA was shown to be an Sp1 antagonist, with strong inhibitory effects on viability and proliferation of myeloma cells [51]. Furthermore, LicA inhibited the growth of oral squamous cell carcinoma cells through Sp1-mediated apoptosis [42]. In RCC, Sp1 can inhibit the expression of vascular endothelial growth factor, by repressing von Hippel–Lindau-mediated transcription [54]. Another study showed that microRNA-429, which suppresses Sp1, could inhibit cell proliferation, migration, and inva-sion in A498 and 786-O RCC cells [55]. From these studies, Sp1-mediated downstream protein expression could modulate multiple signaling pathways that regulate tumorigenesis and metastasis of multiple cancers, including RCC. In this study, we found that LicA could downregulate Sp1 expression and its subsequent binding to the LC3B promoter. This results in enhanced expression of LC3B and the suppression of migration and invasion in RCC cells.

## 5. Conclusions

**Figure 8:**
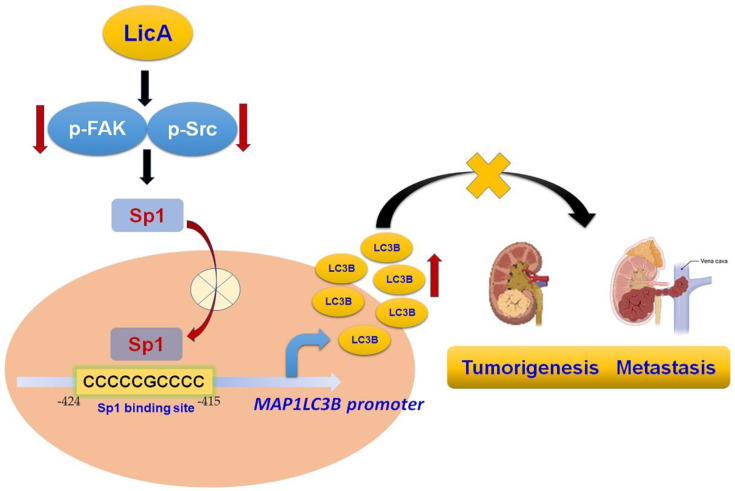
LicA inhibits RCC tumorigenesis and metastasis via the downregulation of the phosphoryl-Src/-FAK pathway. It does so by decreasing the Sp1 transcription activity, resulting in suppressed LC3B expression.

Multiple novel therapeutic regimens targeting signaling pathways in human RCC were the research focus. This is the first study to find that LicA could have potent anti-proliferative and anti-metastatic effects on malignant RCC cells through decreased phosphorylation of Src/FAK, inhibited expression of Sp1, and increased expression of LC3B in succession (Figure 8), highlighting the potential of LicA to lead to new therapies for advanced or metastatic RCC.

## Data Availability

Not applicable.

## Supplementary Materials

The following supporting information can be downloaded at: https://www.mdpi.com/article/10.3390/pharmaceutics15020684/s1. Figure S1: Effects of LicA on apoptosis of RCC cells (786-O, ACHN) and normal HK2 cells. RCC cells (786-O, ACHN) and normal HK2 cells were treated with various concentrations of LicA (0, 6.25, 12.5 and 25 µM) for 24 h. Cell apoptosis was determined by Annexin V-P and PI double staining by flow cytometry. Data are presented as the mean ± SD of at least three independent experiments.
