# Supplementary material for: Licochalcone A Suppresses Renal Cancer Cell Proliferation and Metastasis by Engagement of Sp1-Mediated LC3 Expression
Source: Pharmaceutics. 2023 Feb 17;15(2):684. doi: 10.3390/pharmaceutics15020684 (PMC9966374; doi:10.3390/pharmaceutics15020684)
Supplement: Supplementary file 1 [file pharmaceutics-15-00684-s001.zip › pharmaceutics-2169661-supplementary.pdf]

Licochalcone A suppresses renal cancer cell proliferation and metastasis by engagement of Sp1-mediated LC3 expression

Tsai-Yi Tseng, Chien-Hsing Lee, Hsiang-Lin Lee, Chien-Yu Su, Cheng-Yen Kao, Jen-Pi Tsai, Yi-Hsien Hsieh

Supplementary Figure S1

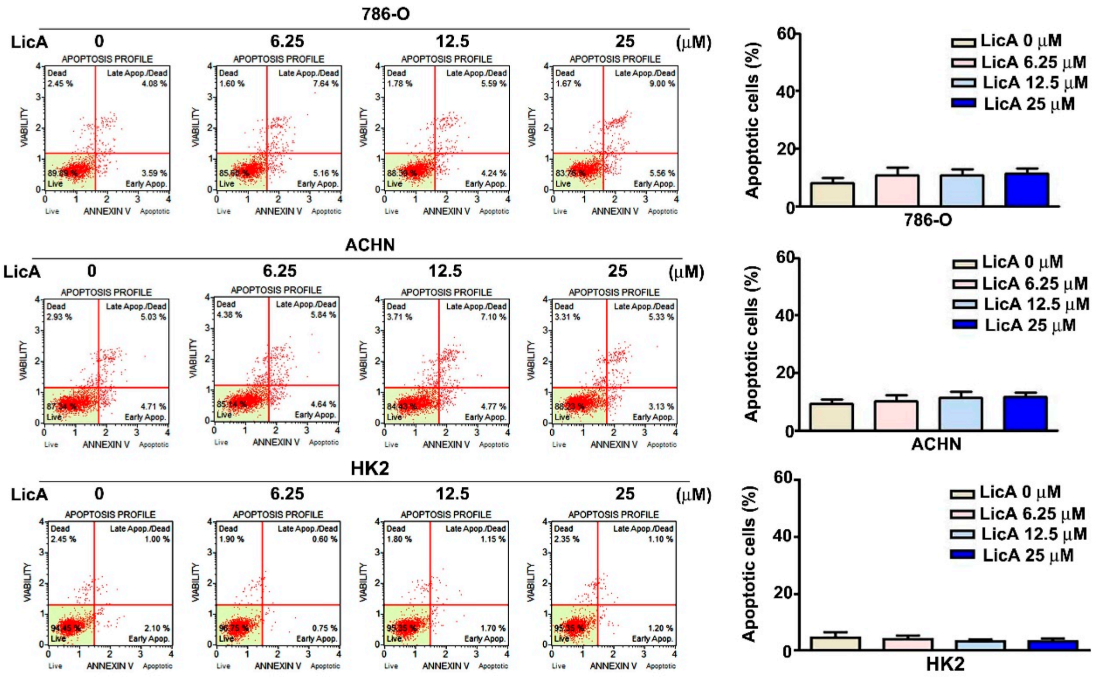

Supplementary Figure S1: **Effects of LicA on apoptosis of RCC cells (786-O, ACHN) and normal HK2 cells**. RCC cells (786-O, ACHN) and normal HK2 cells were treated with various concentrations of LicA (0, 6.25, 12.5 and 25 μM) for 24 h. Cell apoptosis was determined by Annexin V-P and PI double staining by flow cytometry. Data are presented as the mean ± SE of at least three independent experiments.
